# Correction: *Aedes albopictus* microbiota: Differences between wild and mass-reared immatures do not suggest negative impacts from a diet based on black soldier fly larvae and fish food

**DOI:** 10.1371/journal.pone.0301573

**Published:** 2024-03-27

**Authors:** Carlo Polidori, Andrea Ferrari, Luigimaria Borruso, Paola Mattarelli, Maria Luisa Dindo, Monica Modesto, Marco Carrieri, Arianna Puggioli, Federico Ronchetti, Romeo Bellini

The following information is missing from the Acknowledgements section: This work was supported by the Open Access Publishing Fund of the Free University of Bozen-Bolzano.

The correct Acknowledgements is as follows: We thank Fabrizio Balestrino for his assistance in initiating the study. This work was supported by the Open Access Publishing Fund of the Free University of Bozen-Bolzano.
