# Supplementary figures and images for: Aedes albopictus microbiota: Differences between wild and mass-reared immatures do not suggest negative impacts from a diet based on black soldier fly larvae and fish food
Source: PLoS One. 2023 Sep 26;18(9):e0292043. doi: 10.1371/journal.pone.0292043 (PMC10521979; doi:10.1371/journal.pone.0292043)

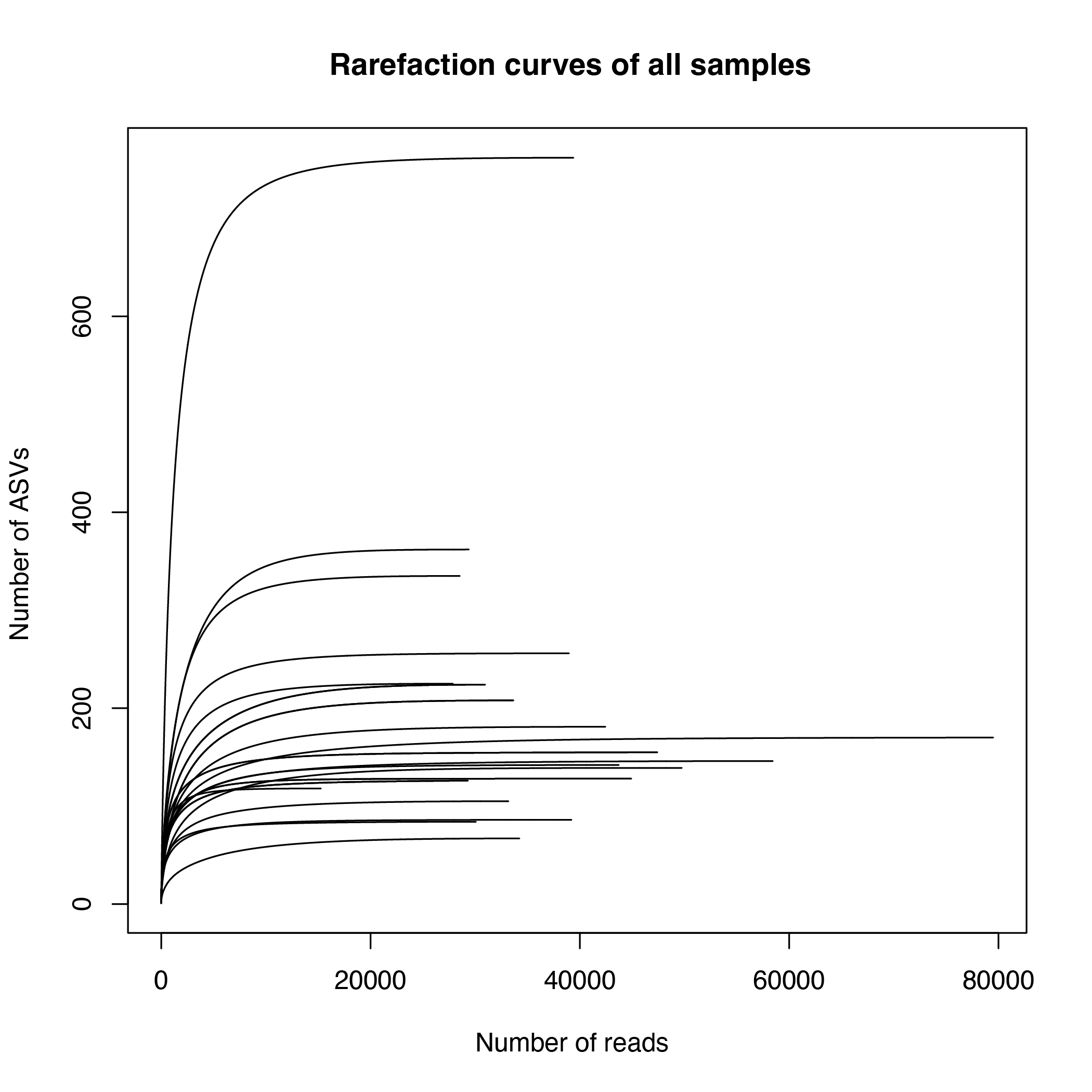

Supplement: S1 Fig — (TIF) [file pone.0292043.s001.tif]

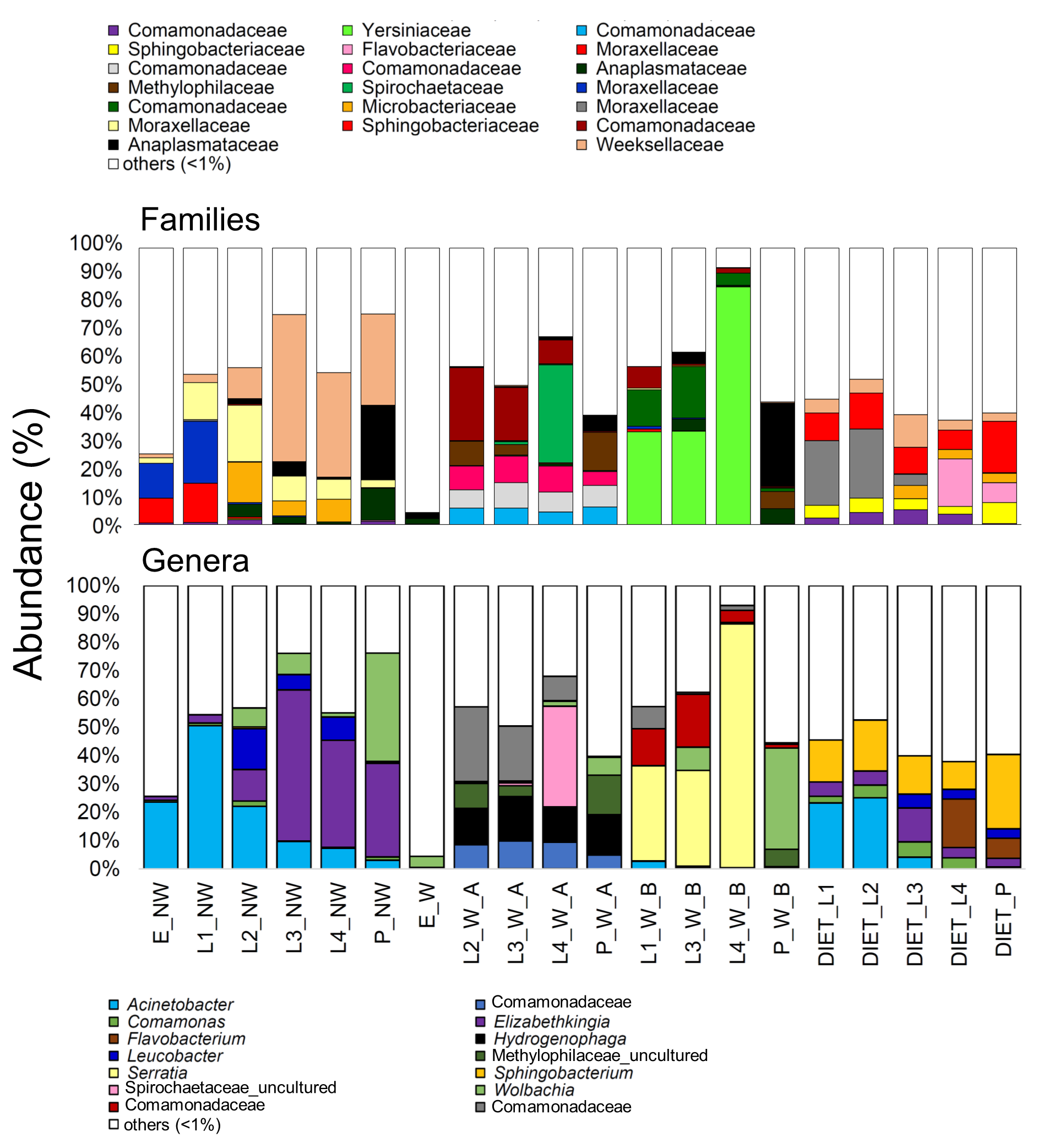

Supplement: S2 Fig — Genera are ranked from left to right from the highest to the lowest effect on group discrimination observed in the NMDS. W: Sample collected in the field (wild); NW: Lab-reared sample (non-wild). (TIF) [file pone.0292043.s002.tif]

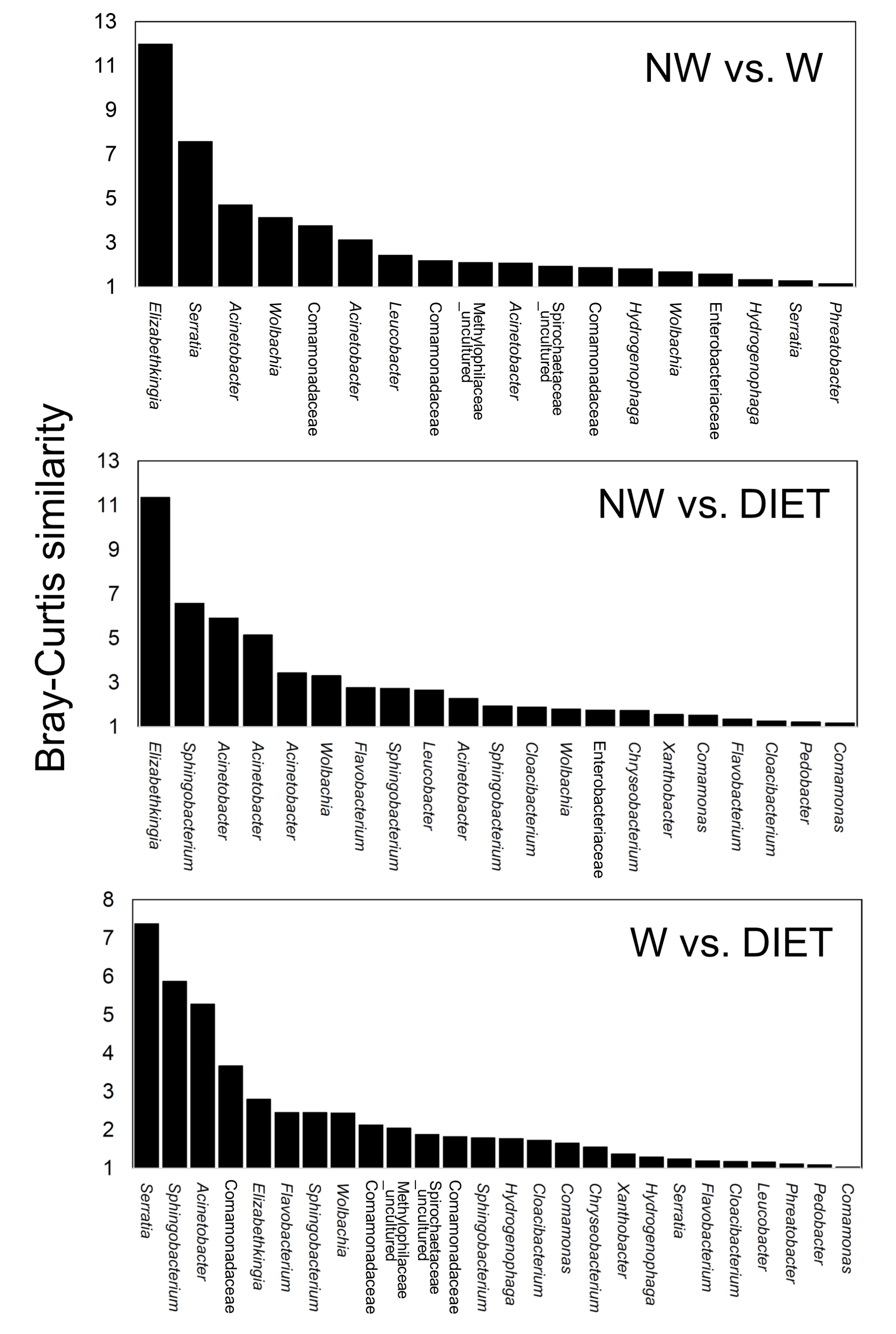

Supplement: S3 Fig — Taxa represented with less than 1% of the total abundance were grouped in the category “other”. L: Larvae, P: Pupae, E: Eggs. W: Sample collected in the field (wild); NW: Lab-reared sample (non-wild). (TIF) [file pone.0292043.s003.tif]

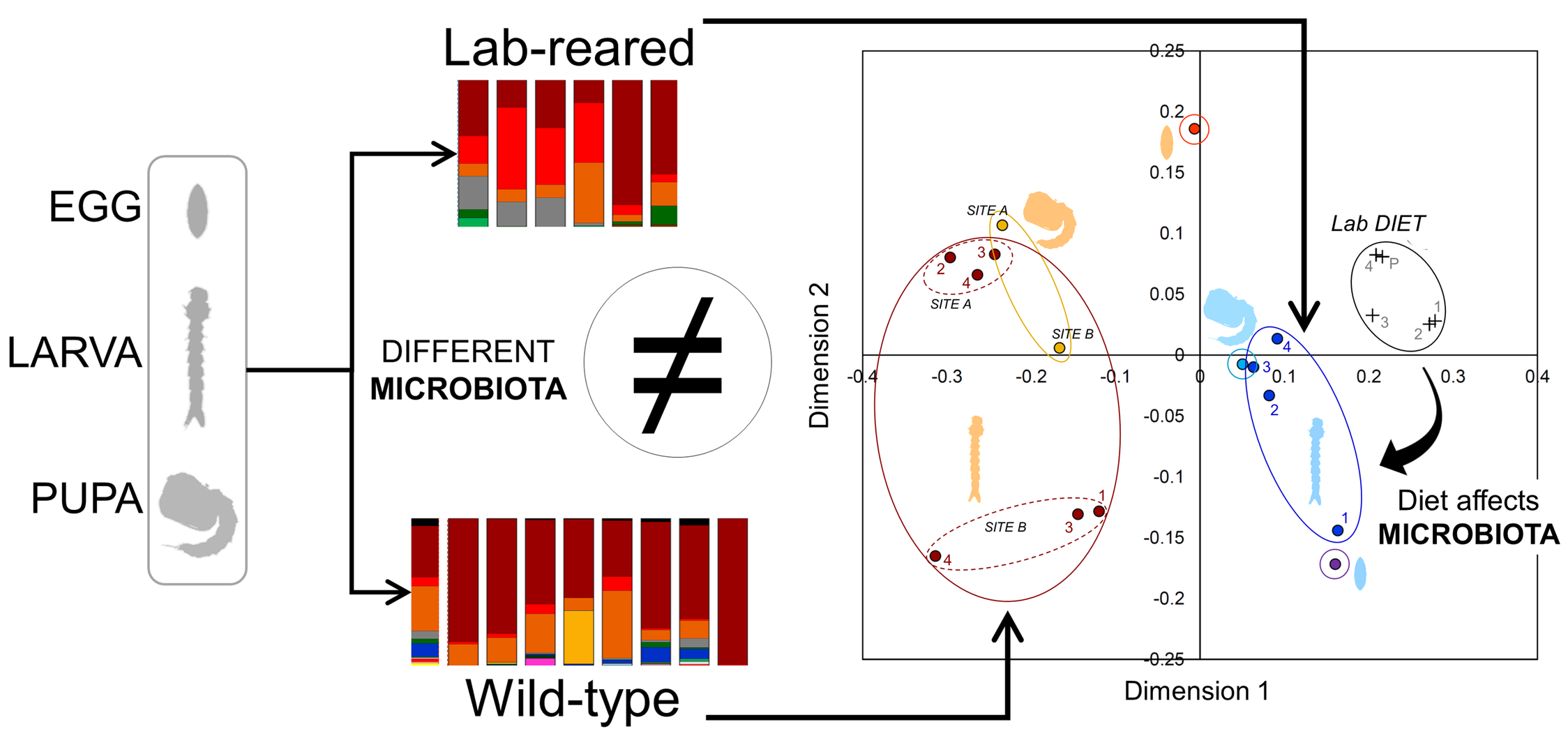

Supplement: S1 Graphical abstract — (TIF) [file pone.0292043.s007.tif]
