# Supplementary material for: Aedes albopictus microbiota: Differences between wild and mass-reared immatures do not suggest negative impacts from a diet based on black soldier fly larvae and fish food
Source: PLoS One. 2023 Sep 26;18(9):e0292043. doi: 10.1371/journal.pone.0292043 (PMC10521979; doi:10.1371/journal.pone.0292043)
Supplement: S1 Table — (DOCX) [file pone.0292043.s004.docx]

**Supporting information**

**S1 Table.** Samples from eggs, larvae, and pupae of analyzed mosquitoes and yield of DNA extracted.

| **Samples from*^$^** | **Biological material** | | mg | | DNA extracted  (ng/ul)** | ratio  (260/280)** |
| --- | --- | --- | --- | --- | --- | --- |
| **NW** | | | | |  |  |
|  |  | |  | |  |  |
| Eggs (E_NW) | about 3250 eggs | | 25 | | 17.4±4.38 | 1.94±0.01 |
| L1_NW | about 100 larvae | | 25 | | 14.40±4.94 | 2.19±0.04 |
| L2_NW | about 40 larvae | | 25 | | 62.50±10 .60 | 2.27±0.09 |
| L3_NW | 10 larvae | | 25 | | 77.50±10.60 | 2.05±0.07 |
| L4_NW | 8 larvae | | 25 | | 84.66±8.38 | 2.10±0.04 |
| pupae_NW) | 5 pupae | | 25 | | 69.85±2.05 | 2.05±0.02 |
|  |  |  |  |  |  |  |
| **W** | | | | |  |  |
| Wild eggs (E_W) | about 3250 eggs | | 25 | | 22.95±3.69 | 1.85±0.01 |
| **Catch basin 11 (Site A)** |  |  |  |  |  |  |
| L2_W_A | 2 larvae | | 1 | | 13.60±1.97 | 2.17±0.24 |
| L3_W_A | 3 larvae | | 7 | | 33.20±4.10 | 2.16±0.06 |
| L4_W_A | 6 larvae | | 21 | | 89.70±0.05 | 2.07±0.05 |
| pupae_W_A | 1 | | 3 | | 34.80±33.6 | 2.11±0.07 |
| **Catch basin 13 (Site B)** |  |  |  | |  |  |
| L1_W_B | 24 larvae | | 6 | | 14.90±1.69 | 2.08±0.08 |
| L3_W_B | 3 larvae | | 7 | | 38.10±2.82 | 2.11±0.14 |
| L4_W_B | 7 larvae | | 25 | | 87.70±7.7 | 2.03±0.07 |
| pupae_W_B | 6 pupae | | 25 | | 51.85±0.03 | 2.03±0.03 |
|  |  | |  | |  |  |

*Each sample was prepared in triplicates; ^$^, L1, larvae of instar I; L2, larvae of instar II; L3, larvae of instar III; L4, larvae of instar IV; **, values represent the mean of three replicates ± SD. W: sample collected in the field (wild); NW: lab-reared sample (non-wild).
