# Supplementary material for: Aedes albopictus microbiota: Differences between wild and mass-reared immatures do not suggest negative impacts from a diet based on black soldier fly larvae and fish food
Source: PLoS One. 2023 Sep 26;18(9):e0292043. doi: 10.1371/journal.pone.0292043 (PMC10521979; doi:10.1371/journal.pone.0292043)
Supplement: S2 Table — W: Sample collected in the field (wild); NW: Lab-reared sample (non-wild). (DOCX) [file pone.0292043.s005.docx]

**S2 Table.** Descriptive statistics (% mean values ± SE) of the 21 genera represented by more than 1% in the bacterial communities analysed, with statistical differences among groups (W, NW, DIET). Dunn’s procedure for paired comparisons was performed only for significant overall difference among groups. Note that in few bacterial genera refer to more than one Box-and-whisker plot, because they belong to different SVs. Undetermined genera were named as the family they belong and referred as “uncultured” or “unknown”. W: sample collected in the field (wild); NW: lab-reared sample (non-wild).

| **Genus** | **W (*N* = 9)** | **NW (*N* = 6)** | **DIET (*N* = 5)** | **Kruskall-Wallis test** | **Dunn’s paired comparisons** |
| --- | --- | --- | --- | --- | --- |
| *Acinetobacter* | 0.21 ± 0.20 | 10.42 ± 6.44 | 0.02 ± 0.02 | χ^2^ = 6.39, *P* = 0.017 | W vs. DIET: *P* = 0.78  W vs. NW: *P* = 0.01  NW vs. DIET: *P* = 0.01 |
| *Acinetobacter* | 0.23 ± 0.19 | 15.46 ± 9.55 | 0.03 ± 0.03 | χ^2^ = 5.99, *P* = 0.002 | W vs. DIET: *P* = 0.75  W vs. NW: *P* = 0.01  NW vs. DIET: *P* = 0.01 |
| *Acinetobacter* | 0.00 | 0.28 ± 0.18 | 21.73 ± 11.45 | χ^2^ = 7.04, *P* = 0.008 | W vs. DIET: *P* = 0.002  W vs. NW: *P* = 0.09  NW vs. DIET: *P* = 0.18 |
| *Acinetobacter* | 0.16 ± 0.13 | 16.16 ± 4.92 | 0.25 ± 0.11 | χ^2^ = 13.28, *P* = 0.001 | W vs. DIET: *P* = 0.25  W vs. NW: *P* < 0.001  NW vs. DIET: *P* = 0.03 |
| Comamonadaceae_unknow | 6.70 ± 2.72 | 0.00 | 0.00 | χ^2^ = 2.79, *P* = 0.06 | - |
| Comamonadaceae_unknow | 6.82 ± 3.80 | 0.00 | 0.00 | χ^2^ = 8.56, *P* = 0.003 | W vs. DIET: *P* = 0.006  W vs. NW: *P* = 0.003  NW vs. DIET: *P* = 1 |
| Comamonadaceae_unknow | 12.84 ± 5.85 | 0.00 | 0.00 | χ^2^ = 11.17, *P* = 0.0008 | W vs. DIET: *P* = 0.002  W vs. NW: *P* = 0.001  NW vs. DIET: *P* = 1 |
| *Comamonas* | 0.44 ± 0.42 | 1.60 ± 0.48 | 7.69 ± 2.16 | χ^2^ = 11.41, *P* = 0.0003 | W vs. DIET: *P* < 0.001  W vs. NW: *P* = 0.007  NW vs. DIET: *P* = 0.25 |
| *Elizabethkingia* | 0.81 ± 0.52 | 35.39 ± 12.15 | 13.45 ± 4.15 | χ^2^ = 14.31, *P* = 0.0008 | W vs. DIET: *P* = 0.005  W vs. NW: *P* < 0.001  NW vs. DIET: *P* = 0.68 |
| *Flavobacterium* | 0.00 | 0.12 ± 0.11 | 12.60 ± 8.85 | χ^2^ = 3.82, *P* = 0.03 | W vs. DIET: *P* = 0.01  W vs. NW: *P* = 0.20  NW vs. DIET: *P* = 0.21 |
| *Hydrogenophaga* | 5.03 ± 2.14 | 0.00 | 0.00 | χ^2^ = 2.79, *P* = 0.06 | - |
| *Hydrogenophaga* | 6.81 ± 2.77 | 0.00 | 0.00 | χ^2^ = 8.56, *P* = 0.003 | W vs. DIET: *P* = 0.005  W vs. NW: *P* = 0.003  NW vs. DIET: *P* = 1 |
| *Leucobacter* | 0.50 ± 0.35 | 7.95 ± 4.26 | 5.88 ± 2.49 | χ^2^ = 2.21, *P* = 0.32 | - |
| Methylophilaceae_uncultured | 8.11 ± 3.96 | 0.00 | 0.00 | χ^2^ = 8.56, *P* = 0.003 | W vs. DIET: *P* = 0.005  W vs. NW: *P* = 0.003  NW vs. DIET: *P* = 1 |
| *Serratia* | 3.30 ± 1.71 | 0.00 | 0.00 | χ^2^ = 1.57, *P* = 0.13 | - |
| *Serratia* | 19.54 ±10.22 | 0.00 | 0.00 | χ^2^ = 2.79, *P* = 0.05 | W vs. DIET: *P* = 0.05  W vs. NW: *P* = 0.04  NW vs. DIET: *P* = 1 |
| *Sphingobacterium* | 0.00 | 0.08 ± 0.07 | 11.29 ± 2.04 | χ^2^ = 11.36, *P* = 0.0004 | W vs. DIET: *P* < 0.001  W vs. NW: *P* = 0.34  NW vs. DIET: *P* = 0.005 |
| *Sphingobacterium* | 0.00 | 0.10 ± 0.10 | 27.04 ± 4.81 | χ^2^ = 11.36, *P* = 0.0004 | W vs. DIET: *P* < 0.001  W vs. NW: *P* = 0.34  NW vs. DIET: *P* = 0.005 |
| Spirochaetaceae_uncultured | 6.01 ± 5.77 | 0.00 | 0.00 | χ^2^ = 1.57, *P* = 0.13 | - |
| *Wolbachia* | 7.82 ± 5.17 | 4.58 ± 2.41 | 0.00 | χ^2^ = 10.75, *P* = 0.004 | W vs. DIET: *P* = 0.003  W vs. NW: *P* = 0.85  NW vs. DIET: *P* = 0.003 |
| *Wolbachia* | 14.66 ± 7.96 | 7.85 ± 5.58 | 0.02 ± 0.01 | χ^2^ = 7.14, *P* = 0.03 | W vs. DIET: *P* = 0.008  W vs. NW: *P* = 0.59  NW vs. DIET: *P* = 0.05 |
